# Ultrasound-Guided Radiofrequency Ablation of Chemodectomas in Five Dogs

**DOI:** 10.3390/ani11102790

**Published:** 2021-09-24

**Authors:** Pablo Gómez Ochoa, María Dolores Alférez, Ignacio de Blas, Telmo Fernendes, Xavier Sánchez Salguero, Beatriz Balañá, Antonio Meléndez Lazo, Alicia Barbero Fernandez, Domenico Caivano, Francesca Corda, Andrea Corda

**Affiliations:** 1 VetCorner, 50012 Zaragoza, Spain; pablogomezochoa@gmail.com; 2 Department of Animal Pathology, University of Zaragoza, 50013 Zaragoza, Spain; deblas@unizar.es; 3 Imaginologia Veterinaria do Porto, 4490-479 Porto, Portugal; insidevetecografia@gmail.com; 4 Department of Animal Science, School of Agriculture, Food Science and Veterinary Medicine (ETSEA), University of Lleida, 25198 Lleida, Spain; xavier.sanchez@udl.cat; 5 Hospital Aralar Veterinarios, Cuarte de Huerva, 50410 Zaragoza, Spain; bboncovet@gmail.com; 6 T-CITO, 46018 Valencia, Spain; contacto@t-cito.com; 7 Department of Veterinary Medicine, University Alfonso X el Sabio, Villanueva de la Cañada, 28691 Madrid, Spain; aliciabarbero.vet@gmail.com; 8 Department of Veterinary Medicine, University of Perugia, 06126 Perugia, Italy; 9 Department of Veterinary Medicine, University of Sassari, 07100 Sassari, Italy; francescacorda91@tiscali.it (F.C.); andreacorda@uniss.it (A.C.)

**Keywords:** thermal ablation, radiofrequency ablation, tumor, chemodectoma, aortic body tumor, ultrasound, ultrasound guidance, canine

## Abstract

**Simple Summary:**

Chemodectomas are rare tumors in dogs. Although brachycephalic breeds seem to be overrepresented, likely due to chronic hypoxia, also non-brachycephalic dogs may be affected. The growth of chemodectomas sometimes leads to compression and/or pericardial effusion; metastases appear infrequently. Therapeutical management includes chemotherapy, surgery, stent placement and radiotherapy. This study describes a novel approach using a minimally invasive technique based on percutaneous ultrasound-guided radiofrequency ablation in five dogs affected by symptomatic chemodectoma. Under general anesthesia and by sonographic guidance, a Leveen electrode, with an umbrella-like configuration, was placed inside the tumor. An alternating current through the electrode produced a spherical volume which increased the temperature, producing tissular necrosis. No complications either during the procedure or in the following days were reported in the five dogs. A rapid clinical improvement was achieved in all the dogs. Changes in the apparent size and echotexture of the mass were also observed. Thermal ablative therapy represents a potential new approach in the clinical management of aortic body tumors.

**Abstract:**

Chemodectomas are low prevalence tumors with complex clinical management. Many present as an incidental finding however, in other dogs, they produce pericardial effusion and/or compression, leading to the appearance of severe clinical signs. There are currently several approaches: surgery, radiotherapy, stent placement and chemotherapy. This is the first description of percutaneous echo-guided radiofrequency ablation of aortic body tumors. This minimally invasive treatment is based on high frequency alternating electrical currents from an electrode that produces ionic agitation and generates frictional heat, causing coagulation necrosis. Five dogs with an echocardiographic and cytological diagnosis of chemodectoma underwent percutaneous echo-guided radiofrequency ablation. At the time of presentation, all the dogs showed clinical signs, such as ascites and/or collapse. There were no complications either during the procedure or in the following 24 hours. Rapid clinical improvement associated with a reduction in size and change in sonographic appearance of the mass were achieved with no complications. Six months follow-up was carried out in all dogs. A second percutaneous echo-guided RFA was performed eight months after the first procedure in one dog. Based on our experience, radiofrequency ablation seems to be a feasible and safe technique, making it a potential alternative therapeutic approach in the clinical management of aortic body tumors leading to severe clinical compromise.

## 1. Introduction

Cardiac tumors in dogs present an incidence ranging from 0.19 to 3% [1,2,3]. Chemodectomas (CDs) are aortic body tumors arising from chemoreceptors in the aortic wall. The term chemodectoma (CD) derives from the Greek words, chemia (infusion), dechcsthai (to), and oma (tumor) [4]. Aortic bodies are related to changes in the chemical constitution of arterial blood, which is stimulated by decreased arterial oxygen tension, increased acidity, increased carbon dioxide tension, increased blood temperature, and certain drugs [5,6]. CDs are overrepresented in brachycephalic breeds, and a higher risk of neoplasia has been reported in English and French bulldogs, Boxers and Boston terriers [7,8]. Chronic hypoxia seems to trigger the development of this neoplasia, as reported in human beings [9,10,11]. Most CDs are detected as an incidental finding during thoracic X-rays or echocardiographic examinations. Clinical signs depend on whether the mass causes compression of adjacent structures, pericardial effusion or seldom chamber invasion. Reported clinical manifestations include ascites, pleural and pericardial effusion, cranial vena cava syndrome, exercise intolerance, cough, lethargy, anorexia, inappetence, collapse, and arrhythmias [5,6,12,13,14,15,16].

The therapeutic approach to CDs has changed over time. Surgical excision of the tumor is difficult given its location, vascularization and the clinical and the anatomical requirements of this procedure [17,18]. In dogs with non-resectable masses and clinical signs associated with pericardial effusion and cardiac tamponade, pericardiectomy can be considered as a palliative procedure with a significantly longer survival time (mean ± SD, 661 ± 170 days) than in dogs treated medically (129 ± 51 days) [16].

The complex approach to this tumor is reflected in the multitude of treatment modalities described in the literature, as well as the potential use of several different modalities in the same dog as part of a multimodal approach. Toceranib phosphate [19,20], stereotactic radiotherapy [8,15,21,22] and stent placement to alleviate local vascular compression have been described [23,24].

Radiofrequency ablation (RFA) is a minimally invasive treatment based on alternating high-frequency electrical currents from an electrode that produces ionic agitation and generates frictional heat, thus causing coagulation necrosis [25,26,27]. In human medicine, the efficacy of RFA has been reported in several primary tumors and metastases, with improvements in the mass effect as well as controlling the biological activity of the tumor [28,29,30,31,32,33,34,35,36,37,38,39,40,41]. In veterinary medicine, percutaneous ultrasound-guided RFA has been used successfully to treat functioning parathyroid and thyroid masses in dogs [42,43,44,45] and cats [46], respectively.

To the best of our knowledge, no reports have described the use of RFA in the treatment of CDs in either veterinary or human medicine. This study therefore describes the percutaneous echo-guided RFA of aortic body tumors and the clinical follow-up in five dogs.

## 2. Materials and Methods

Five dogs with an echocardiographic and cytological diagnosis of CD underwent percutaneous echo-guided RFA and six-month follow-up. Procedures were carried out at a veterinary referral centre VetCorner (Zaragoza, Spain). All owners signed an informed consent form before submitting their dogs to the procedure described below. The owners declined other therapeutical options, firstly offered before RFA.

The breed, age, sex, reproductive status, and body weight (BW) of the dogs are reported in Table 1.

At presentation (T0) each dog underwent a complete physical examination, urine and blood collection, abdominal ultrasonography, thoracic X-ray, echocardiography, and echo-guided fine-needle aspiration of the cardiac-base tumor with a 27 G needle. Both echocardiography and abdominal scans were performed with a Mylab Alpha or a MyLab X8 (Esaote, Genova, Italy) equipped with phased array probes ranging from 1 to 9 MHz and a microconvex probe of 8–11 MHz (Esaote, Genova, Italy). Echocardiographic examinations, with simultaneous ECG, were performed according to the recommendations for standard transthoracic echocardiography in dogs [47]. CD was diagnosed by a combination of echocardiographic and cytological findings. 

The percutaneous echo-guided RFA procedure was performed under general anesthesia with the dog placed in right or left lateral recumbence, depending on the mass location. All dogs were premedicated with intravenous methadone 0.2 mg/kg and diazepam 0.25 mg/kg. Induction was carried out with intravenous propofol, and maintenance with inhalant sevoflurane. Capnography, pulse oximetry, indirect arterial blood pressure, body temperature and ECG were monitored during the procedure.

**Figure 1:**
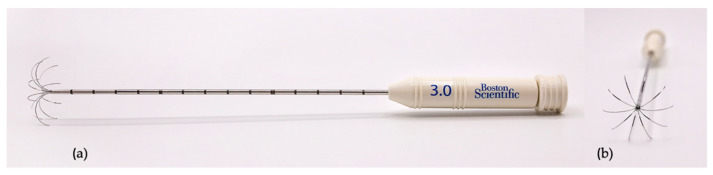
LeVeen Needle Electrode. (**a**) the 3.0 LeVeen Needle Electrode; (**b**) the ten atraumatic umbrella-like tines of the 3.0 LeVeen Needle Electrode.

Thermoablation was performed with an RF 3000 Radiofrequency Generator (Boston Scientific, Malborough-ma, MA, USA) with a LeVeen Needle Electrode (Boston Scientific, Malborough-ma, MA, USA), with an umbrella-like deployment configuration (Figure 1), whose diameter and length were based on the depth and size of the cardiac mass to be ablated [26,27]. 

**Figure 2:**
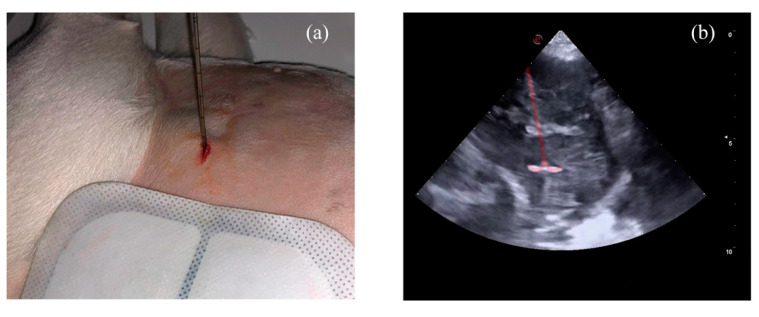
(**a**) LeVeen electrode positioned on the right hemithorax below the grounding pad. (**b**) Sonographic appearance of the electrode deployed within the mass, overimpressed in light red.

Two ultrasound windows, left cranial parasternal and right parasternal short axis views, were mainly used and optimized in order to accurately measure the masses and facilitate the insertion of the electrode. The electrode was positioned in the mass under ultrasonographic guidance from the non-recumbent chest side in a fashion like a routine percutaneous biopsy [48] (Figure 2). The electrode was inserted in the point of contact or in the point of the shortest distance between the mass and the internal thoracic surface. Once inside, ten atraumatic umbrella-like tines were deployed creating a spherical ablation volume (Figure 2. Video S1). At this point the monopolar radiofrequency electrode was activated, resulting in a transfer of electrical current from the tines to the surrounding tissue, leading to coagulation necrosis of the neoplastic tissue [27,49,50].

To disperse the energy produced, two adhesive electrosurgical grounding pads (3M, Saint Paul, MI, USA) were attached to the dorsal region of each hemithorax, providing a safe return path for electrosurgical currents [26].

The time and power of electromagnetic energy tissue exposure were set following the manufacturer’s recommendations. An increasing power algorithm was used until the increase in tissue impedance, which was directly correlated to tissue necrosis, leading to the drop in the power delivered (roll-off point). At this point the thermal ablation was considered complete. The maximum necrosis volume, focused on the compression site, was created without damaging the surrounding tissues [50].

At the end of the procedure, the electrode was removed, and the dog was awakened. Cyanoacrylate tissue adhesive (3M VetBond, 3M Deutschland GmbH, Neuss, Germany) was applied to close the point of entry of the electrode in the skin. Dogs were hospitalized and monitored with ECG and TFAST ultrasonography [51] before being discharged. Clinical and echocardiographic follow-up examinations were carried out one (T1), three (T2) and six months (T3) after the procedure. All the echocardiographic examinations before, during and after the procedure were performed by the same operator (P.G.O.) with several years’ experience in ultrasonography, cardiology, and ultrasound-guided procedures. For each dog the echo settings were kept constant during all the examinations in order to better appreciate the changes in ultrasonographic appearance.

After each RFA procedure, the patients were discharged with anti-inflammatory (prednisone 0.5 mg/Kg, twice a day, orally for one week followed by 0.25 mg/kg, twice a day, orally for 5 days) and antibiotic medication (amoxicillin 12.5 mg/kg, twice a day, orally for one week). Moreover, in order to reduce fibrinolytic activation and minimize the perioperative bleeding risk, one week before and three weeks after the procedure all the dogs underwent treatment with an oral fibrinolysis inhibitor (tranexamic acid 10 mg/kg twice a day) [52,53,54].

## 3. Results

At T0 all the dogs had biochemical, hematological, clotting times (pro- thrombin and activated partial thrombin times) and urinary test results within the normal limits, except for a slight increase in liver enzymes in dogs with ascites (AST 90 U/L and ALT 80 U/L in dog 1; ALT 96 U/L and ALP 216 U/L in dog 3; ALT 106 U/L and ALP 125 U/L in dog 4; AST 85 U/L, ALT 118 U/L and ALP 120 U/L in dog 5; reference intervals AST 10–60 U/L, ALT 10–70 U/L, ALP 0–110 U/L). In all the dogs, the echocardiographic examination showed a rounded mass of homogeneous echotexture, which was slightly hyperechoic, localized in the wall of the ascending aorta at the level of the heart base. Three of the masses compressed the pulmonary artery (dogs 1, 4 and 5), one compressed the caudal vena cava (dog 2), and another one the right atrium, invading the interatrial septum and penetrating the left atrium (dog 3). The three dogs with pulmonary artery compression presented ascites as the main clinical complaint. The two dogs with caudal vena cava and right atrial compression presented syncope as the main clinical sign (dogs 2 and 3). The apparent size of the masses, measured at the compression site, ranged between 4 and 8.5 cm in diameter (Table 2).

**Figure 3:**
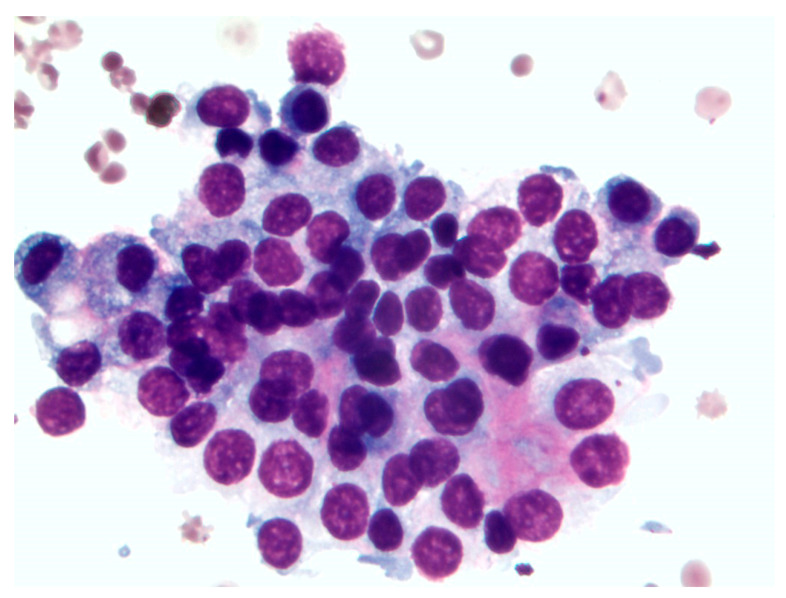
Cytological sample from dog 1.

During echocardiographic examination, sporadic arrhythmic events were observed in two dogs, and included isolated premature complexes of ventricular (dog 3) and supraventricular origin (dog 2). Abdominal ultrasonography and thoracic X-rays showed no evidence of metastasis. In all cases, cytological results showed abundant naked nuclei with a neuroendocrine pattern. Intact cells had rounded edges and a light blue cytoplasm with round to oval nuclei with nucleoli. Anisocytosis and anisokaryosis were mild, and the ratio nucleus/cytoplasm was moderate. The cytological characteristics were compatible with CD (Figure 3). 

Considering the apparent size of the masses, a needle with a maximum diameter of 3 cm was chosen for each procedure, in order not to damage the surrounding tissues [50]. The ablation time until the roll-off point ranged from 11 to 18 min (Table 3). After reaching the roll-off, the electrode was removed from the mass and the procedure was considered completed.

The procedure was fast, with an average time, under general anesthesia, of 35 min, with a mean discharge time of two hours. During the anesthesia, none of the dogs had significant electrocardiographic or hemodynamic abnormalities, and all recovered rapidly. In the two hours following the procedure, no complications such as pneumothorax, bleeding, pericardial effusion, or arrhythmias were detected. No complications were reported by owners during the following 24 h.

One week after the RFA, there was a marked improvement in clinical signs. Ascites disappeared in dog 1, and in dogs 3, 4, and 5 it decreased markedly. Syncopal episodes disappeared in the two dogs who presented them (dogs 2 and 3).

One month after the procedure (T1), echocardiographic examination highlighted a marked reduction in the apparent mass size in all the dogs (Table 3) together with a notable change in the ultrasonographic appearance. The masses became heterogeneous and with mixed echogenicity characterized by multiple hyperechoic and hypoechoic areas absent at T0. Pulmonary artery peak velocity decreased markedly in the three dogs (cases 1, 4 and 5) affected by pulmonary artery compression (Table 3).

**Figure 4:**
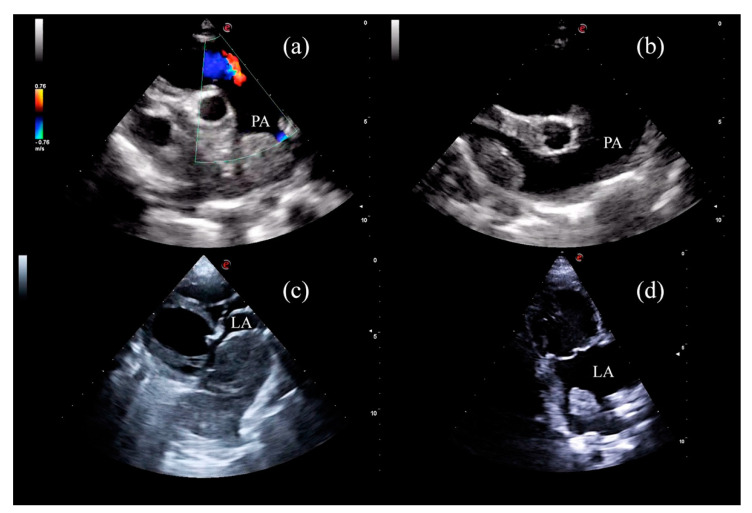
Sonographic appearance before and after the procedure. (**a**) patient 1 before RFA; (**b**) patient 1 after 180 days; (**c**) patient 3 before RFA showing left atrial invasion; (**d**) patient 3 after 180 days. LA (left atrium), PA (pulmonary artery).

Three months after the procedure (T2), the masses remained similar to T1 in echotexture and echogenicity, and showed a slightly decreased in the apparent size in all the dogs (Table 3). Pulmonary peak velocity was stable with no evidence of pulmonary artery compression (Table 3). The CDs apparent size reduction, sonographic changes and the pulmonary peak velocity were maintained six months after the procedure (T3) (Table 3) (Figure 4).

In dog 4, a second percutaneous echo-guided RFA was performed eight months after the first procedure due to a compression of the caudal vena cava and ascites. The RFA was successfully performed, and the dog was still stable six months after the second procedure.

## 4. Discussion

This report demonstrates that percutaneous echo-guided RFA is an effective and safe procedure for treating CDs in dogs with clinical signs associated with this neoplasia. The technique could offer a new and alternative approach in the clinical management of aortic body tumors.

Canine CDs usually present as a single mass growing in the wall of the ascending aorta at the level of the heart base, they are non-functional and generally locally invasive tumors with low metastatic potential [55]. Many dogs present CD as an incidental finding however, in other patients, they produce pericardial effusion or compression of adjacent cardiac structures [55]. In humans heart base CDs are extremely rare tumors and surgical removal is considered the treatment of choice [56,57]. In dogs CDs are low prevalence tumors with a complex clinical management. Several approaches have been described to treat these tumors, ranging from surgery [17], radiotherapy [8,21,22], stent placement [23,24] and chemotherapy [19,20]. There are currently no guidelines indicating which treatment option is best suited for canine CDs however, pericardiectomy and/or surgical excision of masses that are not locally invasive, can be considered the first choice treatment [16,17,55]. According to some authors, surgical excision would be indicated for CDs with a diameter of less than 5 cm [17]. Based on these data and on our experience, we can speculate that the RFA of canine CDs could be a valid alternative approach to the surgical excision in all cases where CDs exceed 5 cm and are locally invasive or in all cases where owners prefer a minimally invasive approach compared to a classic surgery that requires a longer post-operative period.

To the best of our knowledge, this study represents the first description of percutaneous ultrasound-guided RFA of CDs in either veterinary or human medicine. In humans, this technique has been used to alleviate myocardial hypertrophy [58,59,60] and to treat neuroendocrine tumors, such as paragangliomas, in different locations [61,62,63]. Percutaneous ultrasound-guided RFA has been reported for treatment of parathyroid and thyroid masses in dogs [42,43,44,45] and cats [46], respectively.

Unlike other studies, we did not include asymptomatic dogs [21,22]. In this study, all the dogs presented severe clinical signs at presentation. Clinical improvement was evident in all dogs a few days after RFA. In all the dogs, changes in ultrasonographic aspect and a reduction in the apparent size were evident in all follow-up examinations, and most importantly, no growth in the ablated area was observed. It has been demonstrated that fibrosis and scar tissue gradually replace the necrotic area generated by the RFA procedure [64,65]. Therefore, we hypothesized that the sonographic changes were due to the formation of scar tissue and that this had helped to contain the growth of the still active tumoral tissue.

In all the dogs, RFA was proven to be a rapid and safe technique with no complications neither during nor after the procedure. In human medicine, approximately 0.9–5% of patients presented complications directly attributable to this technique, such as infections in the area of necrosis and bleeding [27,66]. To minimize these complications, an antibiotic and fibrinolysis inhibitor were prescribed preventatively. Moreover, prednisolone was prescribed to reduce the inflammatory process due to the procedure (this is the common protocol used in our clinic for these procedures).

No abnormalities or post-RFA damage to adjacent cardiac structures were observed. In human beings, a case of thrombus formation after the use of RFA caterer ablation in atrial fibrillation was described in a patient previously subjected to radiotherapy [10,67]. There are also four references in which the use of RFA to treat atrial fibrillation was linked with the development of myxomas [68,69,70,71]. In our cases, the spherical ablation area produced by the Leveen needle electrode was kept away from the structures unaffected by the tumor, such as the aortic wall, the pulmonary artery wall, and atrioventricular valves.

Placing the electrode into the mass by ultrasound guidance remains the main limitation of this procedure. The procedures described in this report were applied to masses larger than 4 cm, which were in direct contact with or close to the chest wall. Smaller masses or those located in difficult-to-access areas, such as between the right pulmonary artery and the right atrium, may not be treatable by percutaneous ultrasound-guided RFA. Thoracoscopy or thoracotomy could be considered instead of a percutaneous approach, to address small masses in asymptomatic patients or in those with a difficult sonographic window.

It is necessary to emphasize that in our clinic we have performed RFA in 200 cases with various type of tumors from 2015; this could pose a limitation for some clinics with insufficient trained and experienced staff.

The present report has several limitations, firstly the small number of dogs included in the study does not allow us to generalize our results to the entire populations of dogs suffering from clinically relevant cardiac CDs. Secondly, the lack of computed tomography (CT) and magnetic resonance imaging (MRI) examinations, which would probably have been more accurate than ultrasonography in measuring and monitoring the change of shape, size, and texture of the mass after the treatment. However, the owners of the dogs included in the present study refused, mainly for economic reasons, to submit their dogs to serial anesthesia and CT examinations. Thirdly, the lack of Holter monitoring and cardiac troponin measurement to increase the accuracy of ruling out possible arrhythmias and myocardial damage related to the procedure. Finally, another limitation was the lack of long-term follow-up. Since the objective of this study was to assess the safety of the technique and the clinical evolution during 6 months follow-up examinations, a survival study was not set up. This will be carried out in the future by increasing the follow-up time and including more cases.

## 5. Conclusions

Thermal ablative therapy could represent a new and alternative approach in the clinical management of aortic body tumors. Radiofrequency ablation is a feasible, effective, and safe technique. Rapid clinical improvement associated with changes in size and in sonographic appearance of the tumor were achieved with no post-procedural complications. Although further studies are needed to ascertain the role of RFA alone or as an adjuvant technique, it seems promising and we believe it should be considered in the multimodal management of CDs.

## Figures and Tables

**Table 1:** Breed, sex, reproductive status, body weight and age of dogs.

Dog	Breed	Sex and Reproductive Status	BW (kg)	Age (years)
1	Shar pei	F, N	26	12
2	Boxer	M, E	32	10
3	French Bulldog	F, N	18	10
4	French Bulldog	M, N	14	9
5	Spanish Water dog	M, E	22	12

F, female; M, male; N, neutered; E, entire; BW, body weight.

**Table 2:** Mass compression site, apparent size of the mass and main clinical sign at presentation.

Dog	Compression Site	Apparent CD Size at T0 (cm)	Main Clinical Signs at T0
1	Pulmonary artery	6.8	Ascites
2	Caudal vena cava	4.0	Syncope
3	Right atrium with left atrium invasion	8.5	Ascites and syncope
4	Pulmonary artery	7.6	Ascites
5	Pulmonary artery	5.8	Ascites

CD, chemodectoma; T0, before the procedure.

**Table 3:** Compression site, ablation time until impedance roll-off, apparent mass size and peak pulmonary velocity change before (T0) and after the procedure (T1, T2 and T3) in each dog.

			Apparent CD Size (cm)				Pulmonary Artery Velocity (m/s)			
Dog	Compression Site	Ablation Time (min)	T0	T1	T2	T3	T0	T1	T2	T3
1	Pulmonary artery	18	6.8	1.2	0.8	0.8	3.2	1.2	1.1	1.2
2	Caudal vena cava	11	4.0	2.2	1.6	1.3	0.8	0.7	0.7	0.8
3	Right atrium with left atrium invasion	28	8.5	6.5	5.8	5.6	1.1	0.9	0.9	0.9
4	Pulmonary artery	22	7.6	2.4	2.3	2.4	2.8	0.8	0.9	1.0
5	Pulmonary artery	14	5.8	1.6	1.3	1.2	2.2	0.6	0.7	0.7

T0, before the procedure CD, chemodectoma; T0, at presentation; T1, one month after the procedure, T2, three months after the procedure, T3, six months after the procedure.

## Data Availability

Data are available on request to the authors.

## Supplementary Materials

The following are available online at www.mdpi.com/article/10.3390/ani11102790/s1, Video S1: Video S1 shows a LeVeen electrode being positioned within the mass and the ultrasound appearance of the ablated tissue at the end of the procedure.
